# Conditional survival and changing risk profile in patients with gliosarcoma

**DOI:** 10.3389/fmed.2024.1443157

**Published:** 2024-09-06

**Authors:** Lei Xu, Zhihao Yang, Huawei Chen, Chengjun Sun, Chuanjian Tu, Zhiwei Gu, Ming Luo

**Affiliations:** Department of Neurosurgery, Shaoxing Central Hospital, The Central Affiliated Hospital, Shaoxing University, Shaoxing, China

**Keywords:** gliosarcoma, conditional survival, prognosis, nomogram, risk stratification

## Abstract

**Background:**

Conditional survival (CS) considers the duration since the initial diagnosis and can provide supplementary informative insights. Our objective was to evaluate CS among gliosarcoma (GSM) patients and develop a CS-incorporated nomogram to predict the conditional probability of survival.

**Methods:**

This retrospective study using the Surveillance, Epidemiology, and End Results (SEER) database included patients with GSM between 2000 and 2017. The CS was defined as the probability of surviving additional y years after already surviving for x years. The formula utilized for CS was: CS(y|x) = S(y + x)/S(x), where S(x) denotes the overall survival at x years. Univariate Cox regression, best subset regression (BSR) and the least absolute shrinkage and selection operator (LASSO) were used for significant prognostic factors screening. Following this, backward stepwise multivariable Cox regression was utilized to refine predictor selection. Finally, a novel CS-integrated nomogram model was developed and we also employed diverse evaluation methods to assess its performance.

**Results:**

This study included a total of 1,015 GSM patients, comprising 710 patients in training cohort and 305 patients in validation cohort. CS analysis indicated a gradual increase in the probability of achieving a 5-year survival, ascending from 5% at diagnosis to 13, 31, 56, and 74% with each subsequent year survived after 1, 2, 3, and 4 years post-diagnosis, respectively. Following variable screening through univariate Cox regression, BSR, and LASSO analysis, five factors-age, tumor stage, tumor size, radiotherapy, and chemotherapy-were ultimately identified for constructing the CS-nomogram model. The performance of the nomogram model was validated through discrimination and calibration assessments in both the training and validation cohorts. Furthermore, we confirmed that the effectiveness of the CS-nomogram in stratifying GSM patient risk status.

**Conclusion:**

This nationwide study delineated the CS of patients diagnosed with GSM. Utilizing national data, a CS-nomogram could provide valuable guidance for patient counseling during follow-up and risk stratification.

## Introduction

Gliosarcoma (GSM) was initially documented by Strӧebe in 1895, with its comprehensive recognition and understanding advancing following the detailed description by Feigen and Gross in 1955 (1–3). In the 2021 classification by the World Health Organization (WHO), GSM has been categorized alongside epithelioid glioblastoma (GBM) and giant cell GBM as a variant of isocitrate dehydrogenase 1 wild type GBM (4). It constitutes a relatively uncommon malignant brain tumor, comprising approximately 2–8% of all GBM cases (5–7). The latest literatures indicated that GSM may possess neuroradiological, histological, and biomolecular features distinct from those of GBM (8–11). Consequently, there is a need to analyze GSM as a separate subgroup and to develop effective prognostic risk assessment methods and personalized follow-up strategies for these rare patients.

In the clinical management of any malignant disease, an accurate prognostic evaluation aids clinicians in determining optimal treatment strategies and scheduling follow-up appointments effectively (12). In contrast to traditional survival estimates, conditional survival (CS) reflects the evolving nature of survival probability over time (12–15), offering a more meaningful assessment for predicting long-term outcomes in cancer patients who survive beyond a certain period. Additionally, CS analysis for most tumors demonstrated a significant increase in survival rates with longer survival periods (13, 15–17). For extremely poor-prognosis tumors like GSM, utilizing CS prognosis for predicting survival probabilities can provide patients and their families with dynamic and real-time prognostic information, offering them considerable encouragement and hope for survival. In addition to the post-diagnosis period, factors such as tumor characteristics and treatment methods also impact survival probability. Nomograms are constructed based on the most significant predictors of survival (18, 19). Recently, some survival nomograms for GSM patients have been successfully developed (20, 21). However, existing nomograms do not incorporate the duration of a patient’s survival.

**Figure 1:**
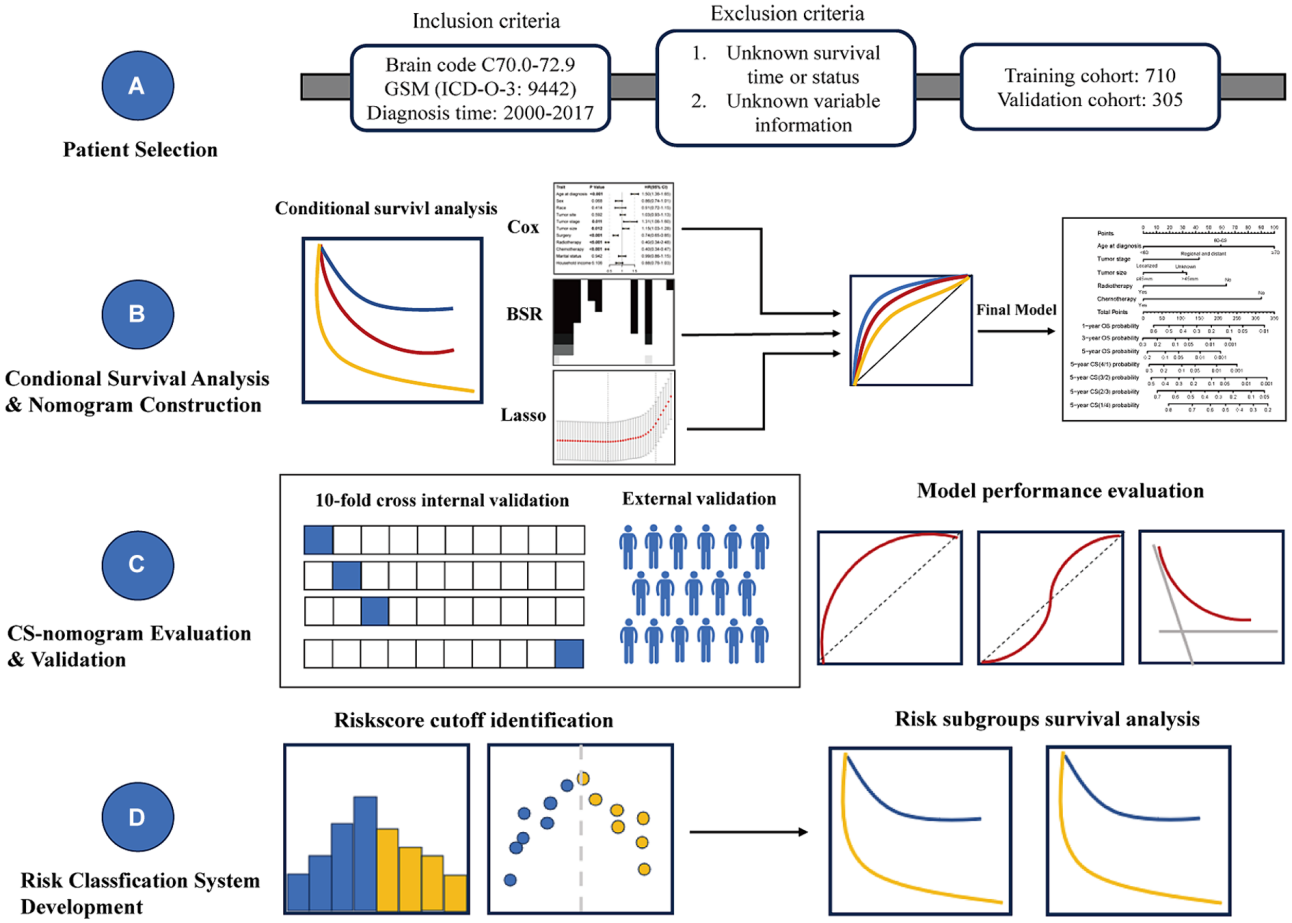
Overview of the research workflow.

Given the low incidence rate of GSM, we utilized the Surveillance, Epidemiology, and End Results (SEER) database to gather GSM cases, aiming to achieve a robust sample size for model stability (22). Furthermore, acknowledging the current lack of CS prognosis analysis for GSM and the persistent necessity for refining prognostic models, we undertook an examination of the present CS status of GSM using the SEER database and we initiated the development of a CS-integrated nomogram model to bridge the existing research gap in this facet of GSM studies (Figure 1).

## Materials and methods

### Patient population

The study was planned as a longitudinal cohort study with a population-based approach, utilizing data retrieved from the SEER database. The SEER program offers comprehensive cancer statistics to mitigate the cancer burden within the US population. As one of North America’s most inclusive tumor registration databases, it stands as a primary source for US cancer statistics, accessible to clinicians worldwide, thus eliminating the need for patient consent.

The inclusion criteria were as follows: (1) Primary site of the tumor: brain codes C70.1-C72.9; (2) Histologic type: GSM (ICD-O-3: 9442); (3) Diagnosis time: 2000–2017.

The exclusion criteria were as follows: (1) Unknown survival time or status; (2) Absence of treatment-related information.

The included variables included age, sex, race, tumor site, tumor stage, tumor size, surgery, radiotherapy, chemotherapy, marital status and household income. Overall survival (OS) was characterized as the duration, in months, from the initial diagnosis of GSM patients to death from any cause by the conclusion of follow-up. The endpoint event was standardized as death from any cause; if this did not occur, censoring was documented.

### Conditional survival concept

Conditional survival, originating from conditional probability in biostatistics, can be computed employing the life-table method. The y-year CS at x years signifies the probability of an additional y-year survival for an individual who has already survived for x years following the initial diagnosis. It is calculated as follows:

CS(y|x) = S(y + x)/S(x) (23).

For instance, in the context of estimating CS for extending survival by 2 years in patients who have already survived 3 years, the calculation of CS(2|3) entails dividing the 5-year Kaplan–Meier survival estimate, denoted as S (5), by the 3-year Kaplan–Meier survival estimate, represented as S (3).

### Model development phase

Among all enrolled patients, we randomly allocated them into training and validation cohorts at a 7:3 ratio for the development and validation of the nomogram. During the preliminary screening for significant prognostic factors, three methods were utilized: univariate Cox regression, best subset regression (BSR), and the least absolute shrinkage and selection operator (LASSO). In the univariate Cox model, factors with a significance level below *p* < 0.05 were selected for subsequent analysis. The BSR method systematically evaluated all feasible variable combinations and determined the optimal variables according to the highest adjusted R^2^ value. Variable selection in LASSO regression was determined based on the lambda.1se value. Subsequently, the final model of the three methods was determined using backward stepwise selection with minimum Akaike’s information criterion (AIC) values and the receiver operating characteristic (ROC) curves with the area under the receiver operating characteristic curve (AUC). The final selected variables were further validated for their prognostic significance and used to develop a CS-incorporated nomogram model via multivariable regression analysis.

### CS-nomogram evaluation and validation

We employed diverse evaluation methods to assess the performance of the model in both the training and validation cohorts. The consistency between the predicted outcomes from the nomogram and the actual observed outcomes was evaluated using a calibration curve generated via bootstrapped resampling. The discrimination of the nomogram was evaluated utilizing ROC curves with AUC values and the concordance index (C-index). Additionally, decision curve analysis (DCA) was performed to illustrate the clinical utility and effectiveness of the CS-integrated nomogram.

### Risk stratification

We further utilized the model to compute the risk score for each patient and analyzed the distribution of risk scores. By identifying the optimal cutoff point for risk scores, we stratified patients from both the training and validation cohorts into high-risk and low-risk groups. Subsequent Kaplan–Meier survival analysis was used to evaluate prognosis between risk groups.

### Statistical analysis

We utilized descriptive statistics to illustrate patient, tumor, and treatment characteristics, while OS was assessed using the Kaplan–Meier method. The data underwent analysis using R software. All statistical tests were conducted as two-sided tests, with a predetermined significance level of *p* < 0.05 to determine statistical significance.

## Results

### Demographic and clinical characteristics of patients

This study included a total of 1,015 records of GSM patients, comprising 710 patients in training cohort and 305 patients in validation cohort. Among all patients, over half of those were aged over 60 years old, constituting 583 individuals, which accounts for 57.4% of the total. Additionally, the male population represented 61.1% of the total cohort. Regarding the tumor characteristics, the vast majority of patients presented with tumors localized predominantly in the supratentorial region (80.2%), demonstrating a localized pattern (83.4%). In terms of treatment, the majority of patients underwent surgical treatment (96.2%), with over half also receiving radiotherapy (74.1%) and chemotherapy (61.0%). Table 1 outlines the baseline clinicopathological characteristics of the patients.

**Table 1:** Clinicopathologic characteristics of GSM patients.

Parameters	Overall (1015)	Training (710)	Validation (305)
Age at diagnosis			
<60	432 (42.6%)	301 (42.4%)	131 (43.0%)
60–69	305 (30.0%)	213 (30.0%)	92 (30.2%)
≥70	278 (27.4%)	196 (27.6%)	82 (26.9%)
Sex			
Male	620 (61.1%)	432 (60.8%)	188 (61.6%)
Female	395 (38.9%)	278 (39.2%)	117 (38.4%)
Race			
White	894 (88.1%)	627 (88.3%)	267 (87.5%)
Non-white	121 (11.9%)	83 (11.7%)	38 (12.5%)
Tumor site			
Supratentorial	814 (80.2%)	567 (79.9%)	247 (81.0%)
Infratentorial	12 (1.2%)	9 (1.3%)	3 (1.0%)
Brain, NOS	189 (18.6%)	134 (18.9%)	55 (18.0%)
Tumor stage			
Localized	847 (83.4%)	597 (84.1%)	250 (82.0%)
Regional/distant	168 (16.6%)	113 (15.9%)	55 (18.0%)
Tumor size			
≤45 mm	422 (41.6%)	281 (39.6%)	141 (46.2%)
>45 mm	448 (44.1%)	326 (45.9%)	122 (40.0%)
Unknown	145 (14.3%)	103 (14.5%)	42 (13.8%)
Surgery			
No surgery	39 (3.8%)	29 (4.1%)	10 (3.3%)
STR	474 (46.7%)	333 (46.9%)	141 (46.2%)
GTR	502 (49.5%)	348 (49.0%)	154 (50.5%)
RT			
No	263 (25.9%)	186 (26.2%)	77 (25.2%)
Yes	752 (74.1%)	524 (73.8%)	228 (74.8%)
CT			
No	396 (39.0%)	289 (40.7%)	107 (35.1%)
Yes	619 (61.0%)	421 (59.3%)	198 (64.9%)
Marital status			
Single	349 (34.4%)	249 (35.1%)	100 (32.8%)
Married	629 (62.0%)	434 (61.1%)	195 (63.9%)
Unknown	37 (3.6%)	27 (3.8%)	10 (3.3%)
Household income			
<65,000$	448 (44.1%)	308 (43.4%)	140 (45.9%)
≥65,000$	567 (55.9%)	402 (56.6%)	165 (54.1%)

STR, subtotal resection; GTR, gross total resection; RT, radiotherapy; CT, chemotherapy.

### Overall and conditional survival

**Figure 2:**
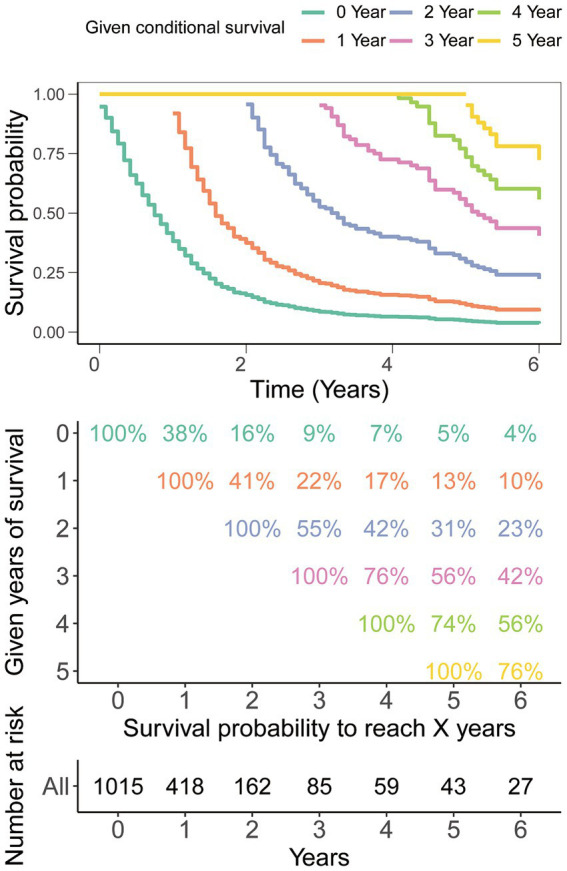
Conditional survival analysis. The survival probability post-diagnosis is depicted in relation to the duration of survival already experienced.

Traditional survival analysis highlighted an exceedingly grim prognosis associated with this tumor type, showcasing a mere 9% survival rate at the 3-year mark and a scant 5% survival rate at the 5-year milestone (Figure 2). However, it is indeed gratifying to report that further analysis using CS demonstrated that the survival probability escalated with each year already survived in relation to the total survival duration. Specifically, the likelihood of attaining a 5-year survival rose incrementally from 5% immediately after diagnosis to 13, 31, 56, and 74% with each additional year survived (i.e., 1, 2, 3, and 4 years post-diagnosis, respectively, Figure 2).

### Construction of CS-based nomogram

**Figure 3:**
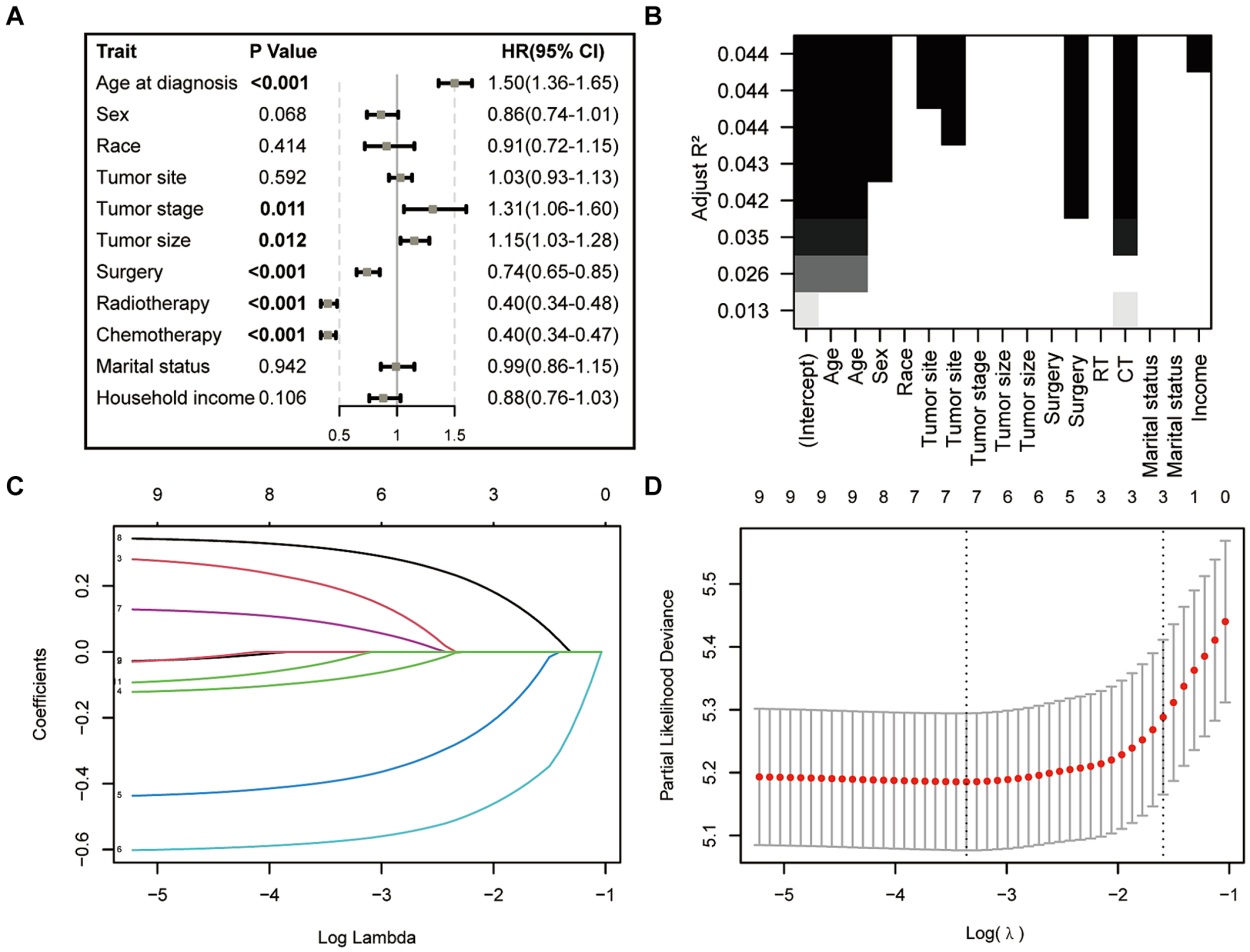
Features selection via univariate Cox regression **(A)**, best subset regression (BSR, **B**), and the least absolute shrinkage and selection operator (LASSO, **C,D**).

**Figure 4:**
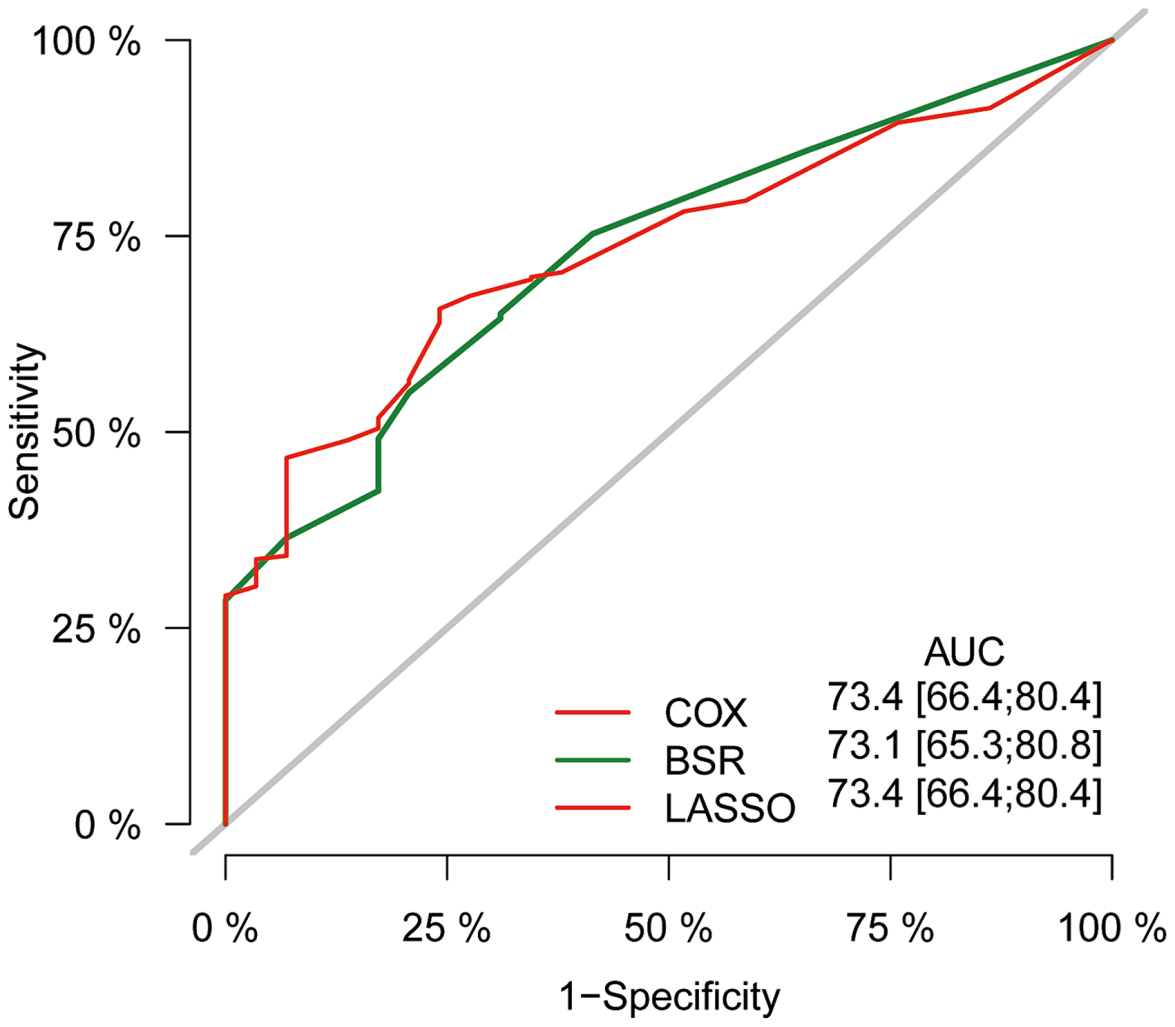
Comparison of AUC values among three models. AUC, the area under the receiver operating characteristic curve.

In the initial stage, we identified six significant features through univariate Cox hazard analysis, including age at diagnosis, tumor size, tumor stage, surgery, radiotherapy, and chemotherapy (as shown in Figure 3A). Subsequently, utilizing the maximum adjusted R2 value from BSR, we pinpointed six variables: age at diagnosis, sex, tumor site, surgery, chemotherapy, and household income (Figure 3B). Furthermore, employing LASSO regression with the lambda.1se value (Figures 3C,D), we identified the following 6 variables: age at diagnosis, tumor stage, tumor size, surgery, radiotherapy, chemotherapy, and household income. Following this, we proceeded with a backward stepwise multivariable Cox regression analysis on the variables of each model in order to discern the final factors. The AIC and AUC values were compared across the three models (Figure 4). In the BSR model, age, surgery, and chemotherapy were identified, resulting in an AIC of 7504.37 and an AUC of 0.731. Conversely, both the univariate Cox and LASSO models identified age, tumor stage, tumor size, radiotherapy, and chemotherapy as significant variables, yielding an AIC of 7478.92 and an AUC of 0.734. Finally, the final model with 5 factors (age, tumor stage, tumor size, radiotherapy, and chemotherapy) were included in the nomogram due to its lowest AIC and highest AUC among three models.

**Figure 5:**
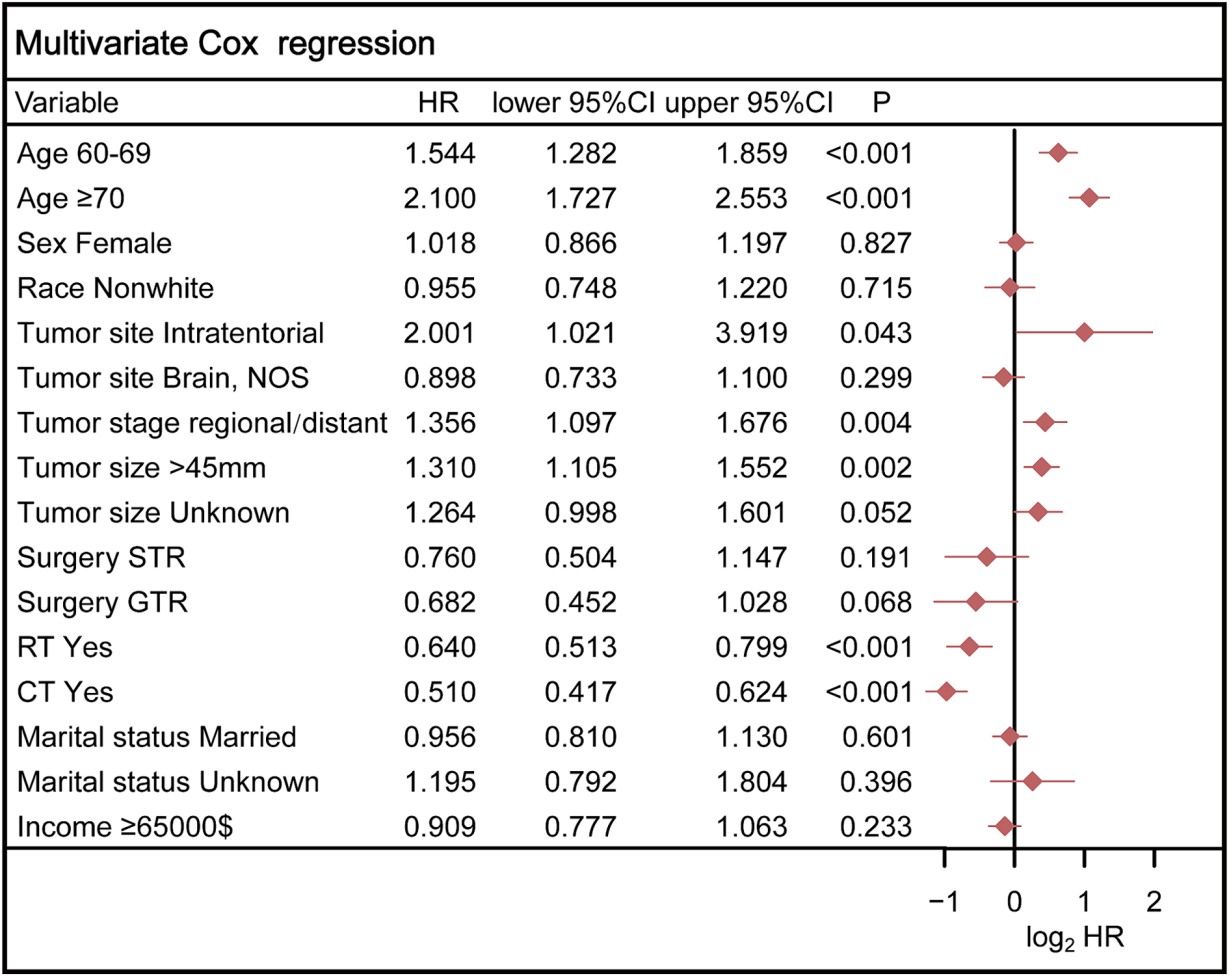
Multivariable Cox regression analysis confirmed the prognostic relevance of these selected variables. STR, subtotal resection; GTR, gross total resection; RT, radiotherapy; CT, chemotherapy.

**Figure 6:**
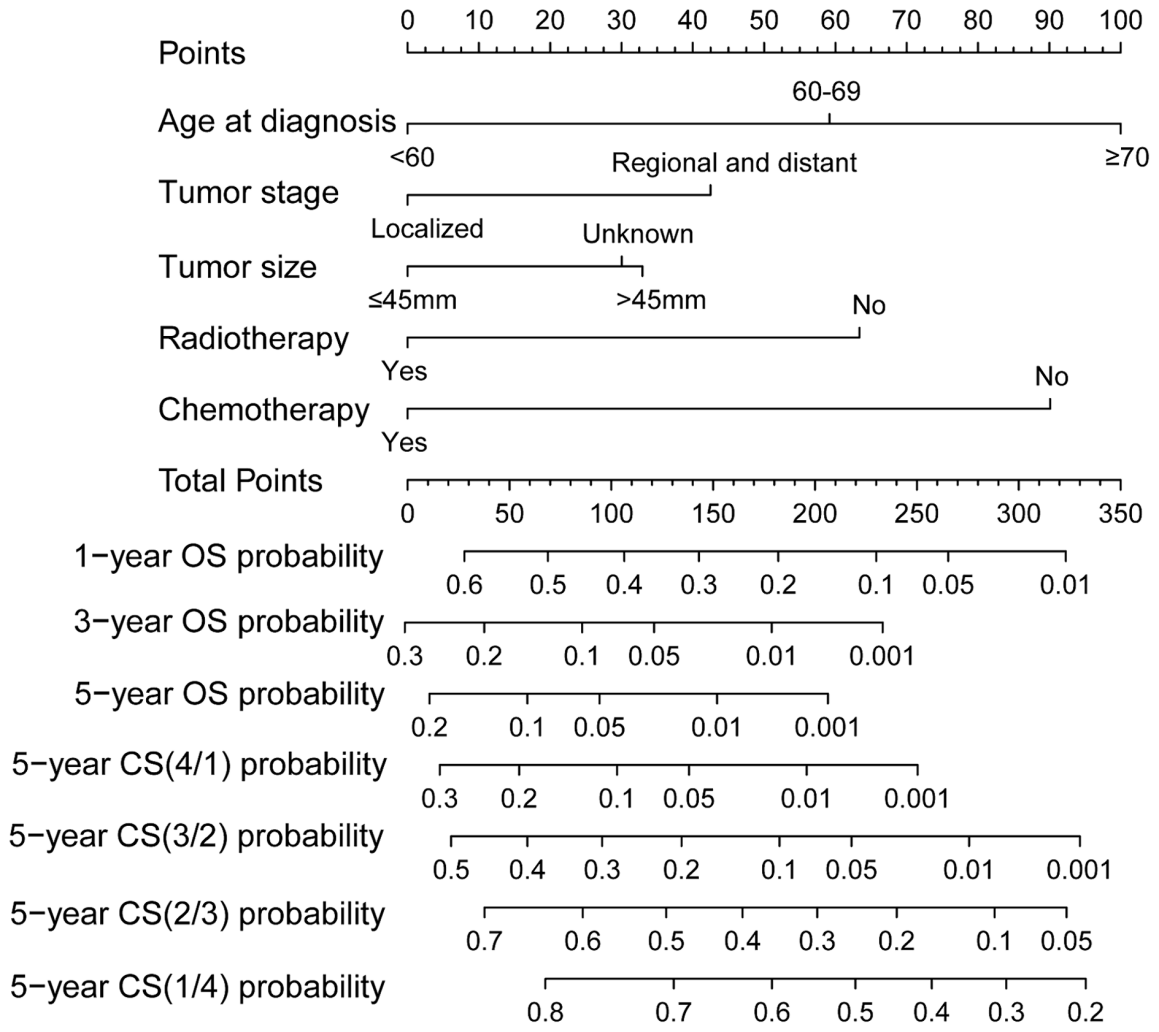
Conditional survival-based nomogram for predicting 5-year conditional survival in patients with GSM.

Ultimately, the selection process favored a model incorporating five factors-age, tumor stage, tumor size, radiotherapy, and chemotherapy-for inclusion in the nomogram. This decision was informed by its demonstration of the lowest AIC and highest AUC compared to the other models. Multivariable Cox regression analysis provided additional validation of the prognostic relevance of these selected variables (Figure 5). Following this, we seamlessly integrated CS into the nomogram model, utilizing these chosen features to effectively construct a CS-based nomogram model for CS prediction (Figure 6).

### Evaluation and validation of the nomogram

**Figure 7:**
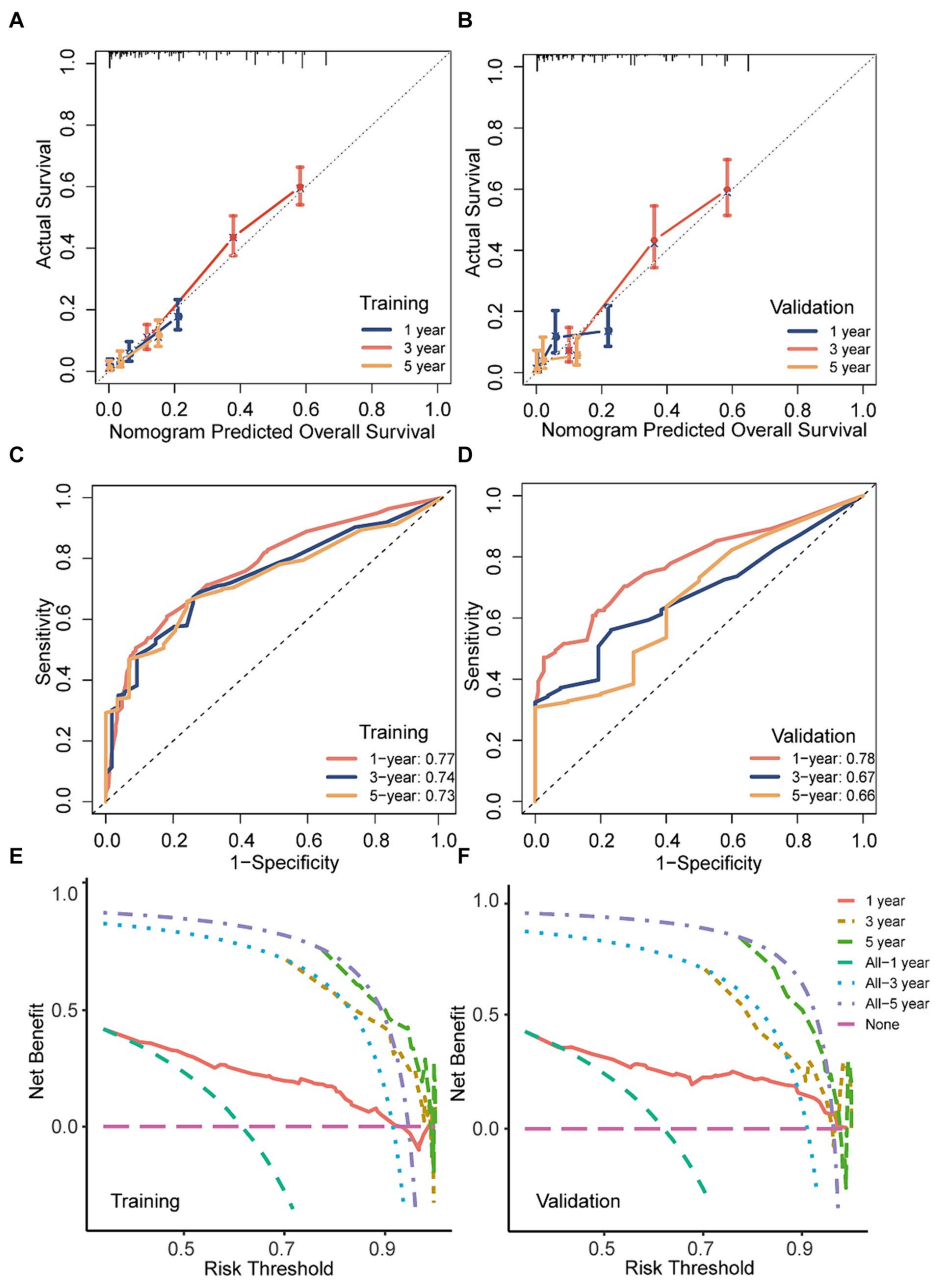
The discriminatory power, calibration, and clinical utility of the nomogram were evaluated in both the training and validation sets. **(A,B)** Calibration curves of the nomogram in both training and validation cohorts; **(C,D)** ROC curves and AUC values of the nomogram in both training and validation cohorts; **(E,F)** decision curve analysis of the nomogram a in both training and validation cohorts.

The performance of the predictive models in both the training and validation cohorts was assessed using various methods, including the calibration plot, c-index, AUC, and DCA. The calibration curves closely aligned with the 45-degree diagonal, indicating robust calibration of the developed nomogram with consistent alignment between observed and predicted probabilities of death (Figures 7A,B). Additionally, the nomogram exhibited promising accuracy in survival prediction, reflected in C-index values of 0.681 and 0.670 for the training and validation cohorts, respectively. ROC curves were employed to evaluate the predictive sensitivity and specificity of the nomogram prediction models. In the training cohort, the AUC values at 1, 3, and 5 years were 0.77, 0.74, and 0.73, respectively, while in the validation cohort, they were 0.78, 0.67, and 0.66, respectively (Figures 7C,D). Furthermore, the DCA illustrated the substantial net benefit of the nomogram in assessing mortality risk, as depicted in Figures 7E,F.

### The effectiveness of the CS-nomogram in stratifying patient risk status

**Figure 8:**
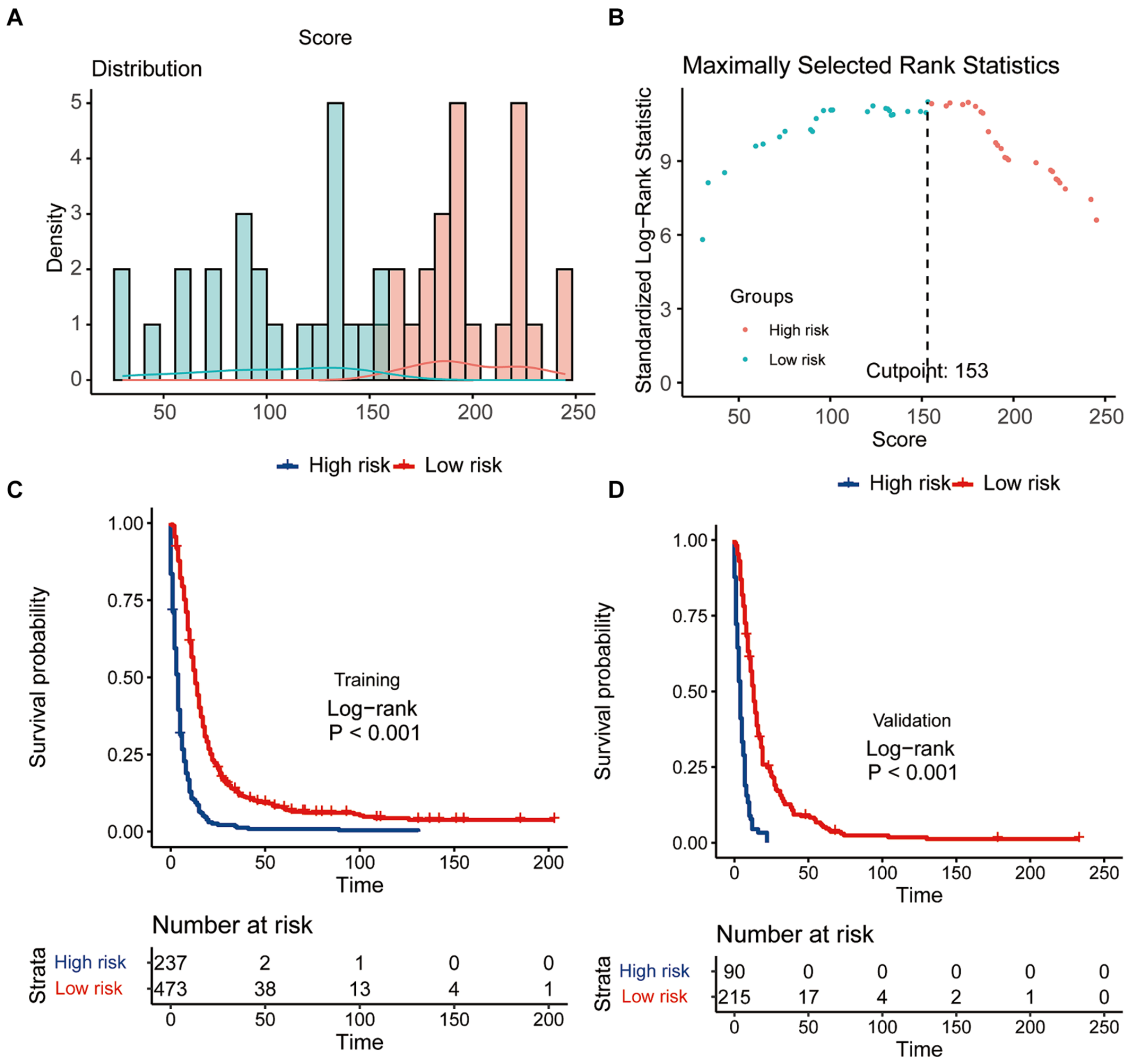
The effectiveness of the CS-nomogram in stratifying patient risk status. **(A,B)** Identification of the optimal cut-off points of patient risk scores. Kaplan–Meier survival analysis with log-rank tests was conducted to assess survival differences among risk groups in both the training **(C)** and validation **(D)** cohorts.

Scores for predictor variables were calculated using the nomogram and then summed to determine the total score for each individual patient. GSM patients were then stratified into low- and high-risk groups according to their nomogram-based scores, employing a threshold value of 153 points (Figures 8A,B). Survival analysis unveiled a significant reduction in the survival probabilities among individuals classified in the high-risk group compared to those in the low-risk group (Figures 8C,D). These findings underscored the potential utility of the CS-nomogram for effectively stratifying risk in GSM patients.

## Discussion

GSM, a highly malignant tumor, is often associated with a low survival rate, leading to pessimistic expectations among patients and their families (24). Therefore, the identification of a gradual increase in survival rates through dynamic prognosis analysis in this study is crucial for instilling confidence in them. This study evaluated CS probability in GSM patients and developed a CS-integrated nomogram, providing both patients and clinicians with precise prognostic information. This graphical tool allows patients to visually perceive that their likelihood of cancer survival improves over time. The model’s excellent performance was further validated through various assessment methods in both training and validation cohorts, highlighting its potential as a valuable tool for real-time dynamic clinical prognosis prediction.

The CS takes into account the duration of a patient’s survival when estimating the probability of continued survival (12–14). The likelihood of attaining a 5-year survival in GSM patients rose incrementally from 5% immediately after diagnosis to 13, 31, 56, and 74% with each additional year survived (i.e., 1, 2, 3, and 4 years post-diagnosis, respectively). The real-time and dynamic presentation of this intuitive prognosis information is expected to alleviate anxiety among GSM patients and their families, greatly bolstering their confidence. Additionally, these real-time dynamic updates will also offer valuable insights for the formulation of patient treatment plans and follow-up strategies. Therefore, CS serves as a valuable adjunct to forecasting post-diagnosis survival in GSM, as also shown in studies in other malignancies.

The final iteration of our CS-nomogram model incorporated age at diagnosis, tumor stage, tumor size, radiotherapy, and chemotherapy. The prognostic significance of age has been confirmed in multiple studies, with older age being associated with poorer outcomes. For tumor characteristics, according to other studies (24), significant differences in the size of GSM were not observed. And the variance in these findings may be attributed in part to differences in sample size. Furthermore, our study indicated that patients at an advanced stage had a poorer prognosis, a finding consistent with another study. In terms of GSM treatment, at present, there exist no universally standardized management protocols for GSM. Generally, maximal surgical resection followed by adjuvant therapy is advised (21, 25). While our analysis did identify a certain association between surgery and prognosis (*p* = 0.068), it seems to contribute less to the prognostic prediction compared to other selected variables. Therefore, to simplify the model, we ultimately included only the current five variables. Another reason for the limited prognostic impact of surgery in this study may be the relatively small number of patients who did not undergo surgery, which could introduce statistical bias into the results. While the efficacy of surgery as a treatment is well-established, future model development should account for larger sample sizes to more accurately assess its prognostic value.

Finally, in view of the existing models established for GSM, Feng et al. similarly constructed a prognostic model for OS in GSM patients, identifying patient age, tumor size, tumor stage, and chemotherapy as significant prognostic factors (21). However, unlike our study, they did not integrate CS into their final model, and radiotherapy was not included in their model. Moreover, existing models concentrated solely on OS and possessed smaller sample sizes relative to our study (20, 21). Our research represents the inaugural endeavor to model CS, furnishing patients with more intuitive, dynamic, and precise prognostic insights, thereby carrying wider applicability and clinical significance.

This study has several limitations. Due to its limitations of SEER database, some variables potentially impacting survival, such as molecular pathological data, were not available and could not be incorporated into our model. Additionally, as a retrospective study, the presence of selection bias was inevitable. Finally, since our model was developed based on a population cohort from the United States, further validation on external cohorts is needed to assess its generalizability.

## Conclusion

This nationwide study delineated the CS of patients diagnosed with GSM. A CS-nomogram derived from national data could offer valuable guidance for patient counseling during follow-up and risk stratification. It is advisable to externally validate the nomogram and CS estimates in additional cohorts of GSM patients.

## Data Availability

Publicly available datasets were analyzed in this study. This data can be found at: https://seer.cancer.gov/data-software/.
